# Arthroscopic Contribution of Synthetic Graft in Tibiotalocalcaneal Arthroscopic Fusions

**DOI:** 10.7759/cureus.12334

**Published:** 2020-12-28

**Authors:** Jaime A Sánchez Lázaro, Óscar Fernández Hernández, Francisco Madera González

**Affiliations:** 1 Orthopedics and Traumatology, Complejo Asistencial Universitario de León, León, ESP; 2 Orthopedics and Traumatology, Integrated Biomedical Engineering & Health Sciences, León, ESP; 3 Surgery, Universidad de Salamanca, León, ESP

**Keywords:** tibiotalocalcaneal arthrodesis, arthroscopic fusion, graft, peptide-15

## Abstract

Nonunion is a frequent complication of tibiotalocalcaneal arthrodesis. The risk of nonunion increases significantly for those patients with systemic comorbidities and smokers. The purpose of this article is to show the proper way to supplement our arthroscopic fusion surgeries with biomaterial (peptide-15) graft. We have achieved an increase in consolidation rates in complex patient cases. We can conclude that this is a simple and reproducible technique.

## Introduction

Tibiotalocalcaneal (TTC) arthrodesis is an aggressive surgery, with high rates of complications including infection, wound dehiscence, malunion, and nonunion [1].

Numerous fixing methods have been developed to try to achieve a stable and pain-free fusion (blocked plates, compression screws, external fixation, retrograde nails). With these methods different approaches have been developed, mainly fixations with plates or nails. Although the former achieves better reduction and greater contact surface, plates require more extensive skin incisions which often exacerbate problems in achieving adequate skin closure in an already challenging anatomical area. In contrast, intramedullary nailing requires smaller incisions with more respect for the skin. Recently, arthroscopic assisted TTC arthrodesis has become a commonly used method for cases without major deformity, particularly in patients at risk of compromised wound healing (diabetes, rheumatoid arthritis, previous surgeries) [2].

Given the large number of complications, different bone grafts and bone substitutes have also been used to improve bone union. However, there are actually few published articles documenting the clinical cases and series supporting the efficacy of many of the bone graft options. The decision to use any kind of bone graft (cancellous autograft, cancellous allograft, structural autograft and structural allograft) or new orthobiologic substitutes, often indicates a more complex procedure or the treatment of high risk patients [3]. In high risk patients, documented nonunion rates are as high as 16% [4], probably due to compromised vascularity and subsequent delivery of nutrients and host reparative cells to the arthrodesis site [5]. Not all bone grafts are practical options for arthroscopic use. However in this technical paper, we describe the use of a biomaterial (i-FACTOR^(R)^ Peptide Enhanced Bone Graft) containing a biomimetic peptide (P-15) which replicates the cell binding domain of Type 1 collagen.

## Technical report

Trial insertion of i-FACTOR Flex FR (a dry, flexible graft strip) was conducted prior to surgery to determine best practice and graft location. Experience was also gained from open surgery performed in high risk patients (also tibiotalocalcaneal arthrodesis). 

**Figure 1:**
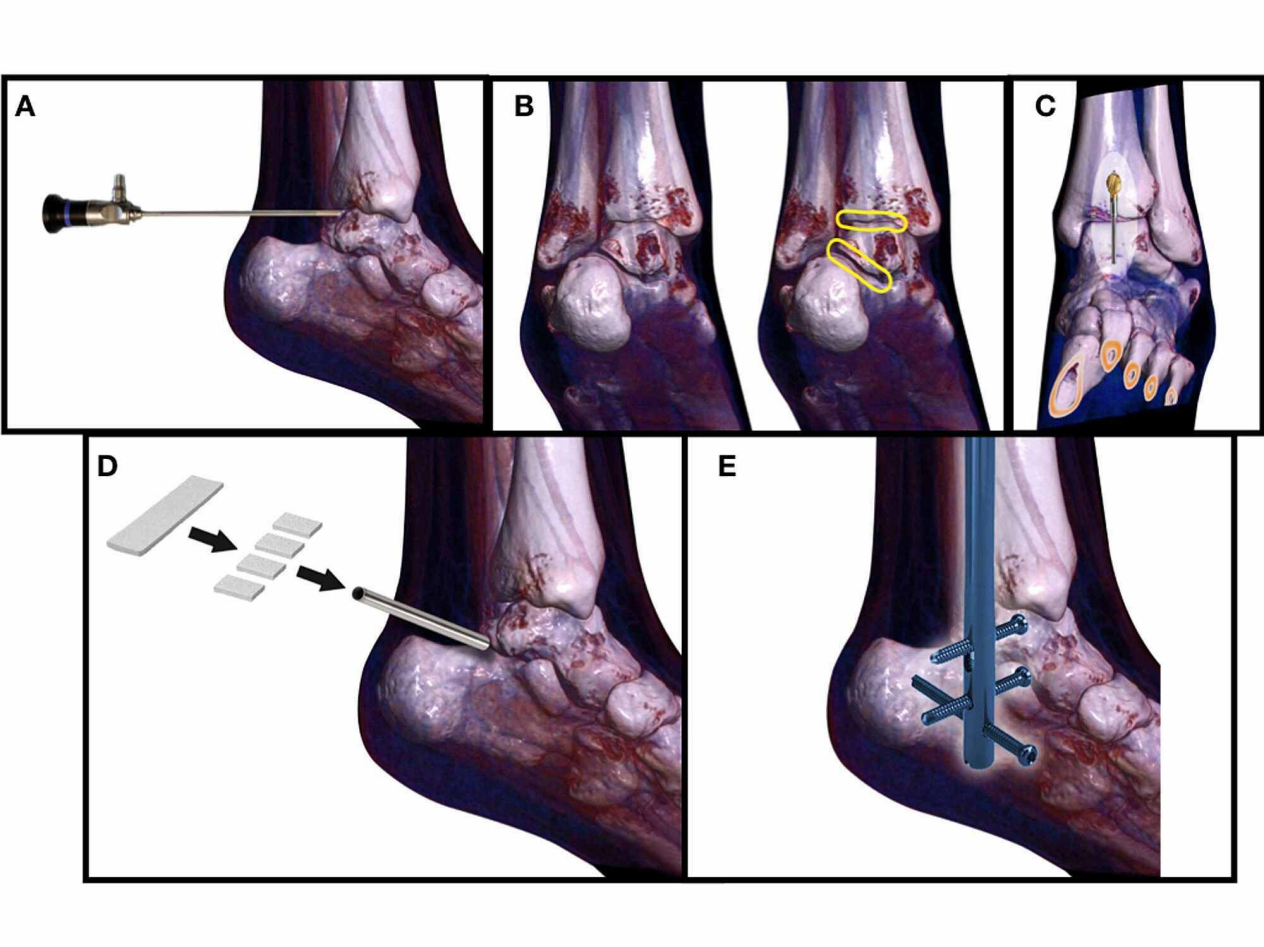
Fusion steps A) arthroscope access, B) preparation of surfaces,C) intramedullary preparation, D) i-Factor^(R)^ inserted into subtalar and tibiotalar joint, E) nail final location.

The procedure was performed under general or spinal anesthesia with the decubitus prone position. Free movement was allowed in the treatment ankle to allow the correct fusion position to be achieved and the contralateral limb was positioned a few centimeters below to allow lateral radiographic visualization. A tourniquet cuff was applied to the thigh (maximum pressure applied 320 mmHg, normally 100-150 mmHg above systolic pressure). Posterolateral and posteromedial portals were used as described previously by van Dijk [6]. Standard arthroscopic instruments were used: 4.5mm x 30º arthroscope, 4.0mm Shaver Blade, 4.0mm burr, low-pressure infusion pump at 35 mm Hg (Stryker, Greenwood Village, CO) together with ring-handle graspers and punches, curettes, osteotomes, elevators and chondral picks (Arthrex, München Germany). We started the artroscopic procedure as published previously by van Dijk and recently by Vilá y Rico 2020 [7]. Crushing of the subtalar and tibiotalar joints was performed with motorized cutters. Arthroscopic control of milling according to the nail diameter and dimensions is an important surgical process which may influence fusion outcomes. As the nail diameter increases, the bone contact area for the arthrodesis decreases at tibiotalar and subtalar joints thereby reducing the space available for fusion. Therefore, it was very important to center the nail in the talus using the arthroscope to allow bone contact around the complete circumference of the nail when the arthrodesis nail is placed (Phoenix®, Zimmer, Warsaw, USA). Before nail compression and locking of the nail, arthroscopic insertion and packing of i-FACTOR Flex FR bone graft was completed between the joint surfaces, subtalar and tibiotalar joints (Cerapedics Inc., Westminster, USA) (Figure 1).

**Figure 2:**
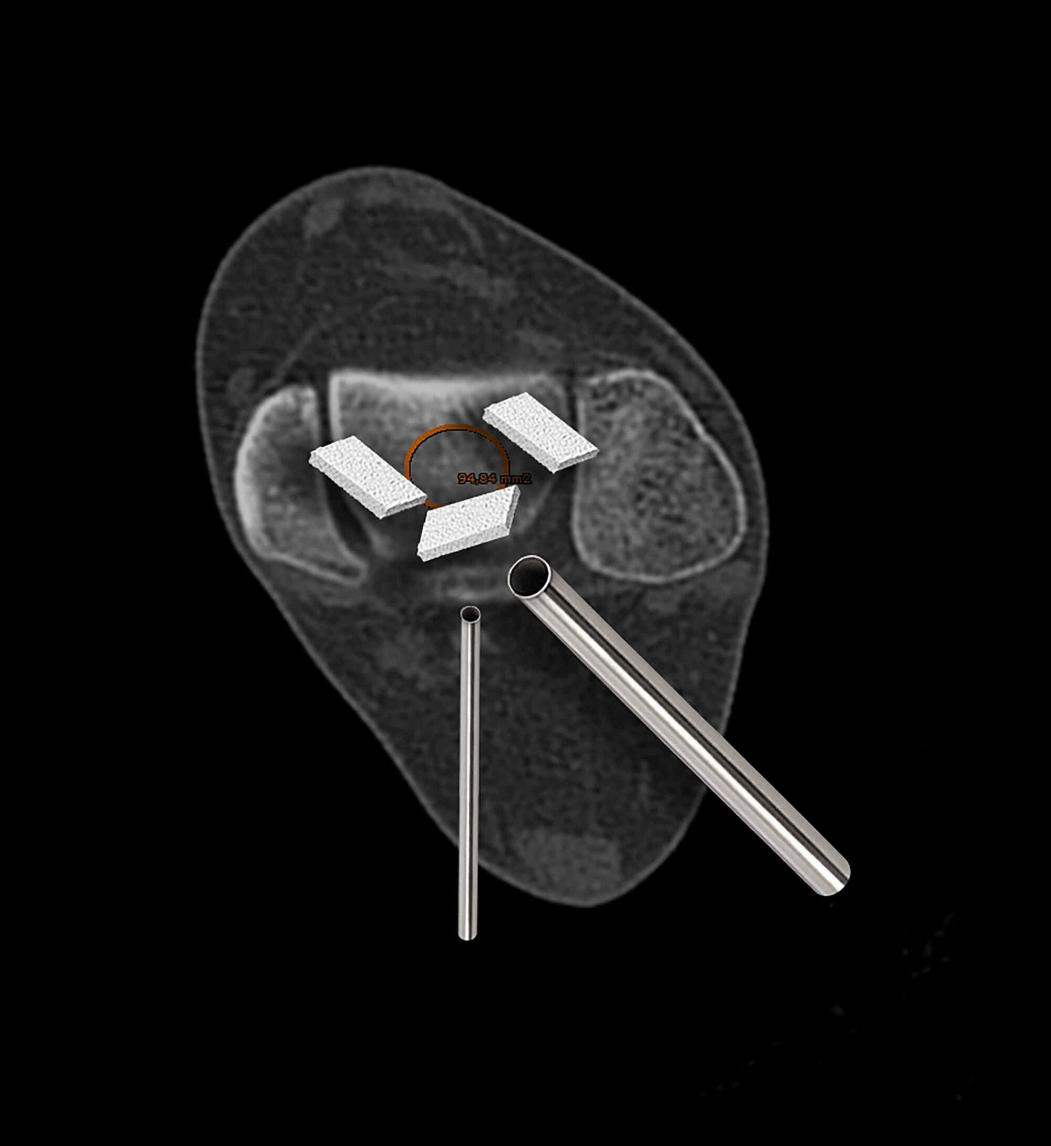
biomaterial graft placement Detail of i-Factor^(R)^ placement around nail

During this procedure, thin dry strips of i-FACTOR^(R)^ Flex FR (approximately 5mm) were cut and pushed through the cannula into the joint space surrounding the nail, into the subtalar and tibiotlar joints (Figure 2).

The procedure was concluded with static locking and compression at the tibiotalar and calcaneus level, following surgical technique provide by nail designers. Wound suture of the arthroscopic portals and nail insertion approach with 2/0 absorbable suture. The average time of all our surgeries was 90 minutes.

**Figure 3:**
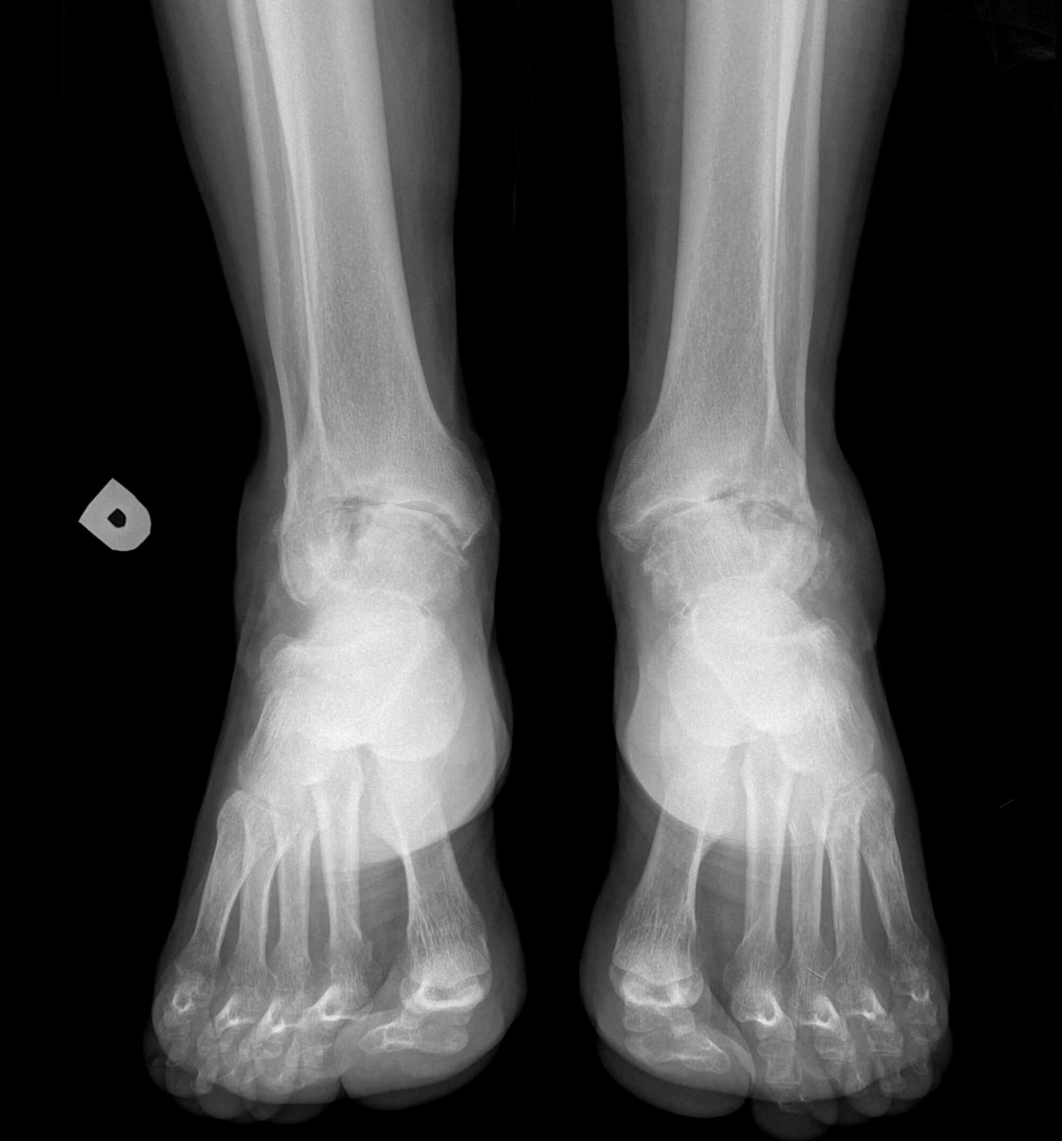
Preoperative radiographs Antero-posterior RX view of severe ankle degeneration, weight bearing RX was not possible due to patient pain

**Figure 4:**
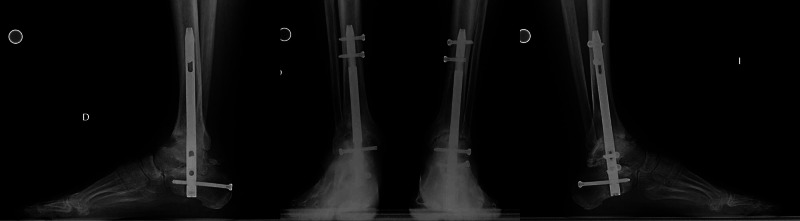
Postoperative weight bearing radiographs Three months after second surgery (right), weight bearing RX

Postoperative management was following our tibio-talo-calcaneal arthrodesis postop protocol: soft bandage for two weeks, no weight wearing allowed until skin wound is correctly healing, followed by progressive partial weight bearing introduced with walker boot at three weeks. At two months crutches were removed. Figure 3 and 4 depict a case report as an example.

This procedure can be used in subtalar arthrodesis in cases with bone defects (Video 1).

**Video 1:** Tibiotalocalcaneal arthroscopic fusion Arthroscopic i-FACTOR^(R)^ inserting and packing.

## Discussion

Non-union rate in tibiotalocalcaneal arthrodesis is high, with a nearly 23% non-union rate reported for the subtalar joint, reaching rates of 31.8% in one of the two joints, tibiotalar and/or subtalar. Nailing is a common option owing to the biomechanically superiority compared with other options. Clinical risk also lies with the long consolidation times for the subtalar (42-734 days) and ankle joints (42-464 days)[1,8].

Apart from the poor fusion rate of these joints due to their own anatomical characteristics, we should not forget the great comorbidity that these patients present, as different systemic pathologies are associated with ankle degeneration (e.g. Charcot disease, rheumatoid arthritis, sequelae of pylon tibial fractures). In addition to these diseases, we must remember that smokers have a nonunion rate up to four times higher than nonsmokers. A published series in patients with risk factors and endomedullary nailling reports up to 45% of nonunion, 38% of major complications and 56% of minor complications. Perhaps among all of them, we can highlight diabetes mellitus which is increasing in Western society, and its complications like the current problematic diabetic foot [9,10].

According to the literature, these percentages can be improved by performing the arthrodesis assisted by an arthroscopic technique, especially in patients at risk in which the infection decreases very significantly [11]. However, this is not the case with range of fusion in which no significant differences are found. The use of bone substitutesis is becoming more frequent with the aim of improving the consolidation rates and, if possible, to reduce the time to consolidation [12-13]. Numerous bone graft options are now available, including PRP (platelet rich plasma), BMP (bone morphogenetic protein), BMA (bone marrow aspirate), DBM (demineralized bone matrix), frozen bone graft (allograft) and autogenous graft (self-grafting) [14]. Autogenous cancellous bone graft remains a reasonable gold standard even with a lack of level I evidence. However, this procedure if often associated with a high rate of complications and postoperative morbidity (up to 23%) after bone harvesting [3,15]. Given that the use of these products incurs an additional cost to surgery and there is no clear guarantee of success, the question arises as to when to provide these substitutes for surgery. In a survey of orthopaedic surgeons carried out in 2013, the majority of respondents agreed that bone grafts should be used in the presence of non-union and/or avascular necrosis [16]. Wallace et al. proposed a decision algorithm for the correct use of these products, advising their application in revision surgeries, surgeries with bone defects, comorbidities and findings that may hinder bone consolidation [14]. Probably, all these graft options can be introduced by arthroscopy. However, due to their physical properties, autologous bone graft and i-Factor^(R)^ are easy ones to introduce by a cannula.

After reviewing the literature, it is clear that the use of orthobiologics in high risk patients is justified. However, which bone graft substitute should be used, after the autograft that possesses properties of osteo-induction, osteo-conduction and osteo-genesis, what do we have?. The use of cellular based allografts is an alternate treatment option which has reported promising results in foot and ankle arthrodesis. However, evidence remains limited for these regenerative therapies involving multipotent adult progenitor cells [3,17]. Demineralized bone matrices (DBMs) and the allografts present osteo-conductive and osteo-inductive properties. However, few of these options have evidence to support their use as a standalone substitute [18]. It is known that bone has a remarkable capacity for growth, regeneration and remodeling. Based on this premise, it seems logical to use grafts that enhance this capacity. In our case we have used i-FACTOR^(R)^ which includes the P-15 biomimetic peptide which has been shown to stimulate osteogenic precursor cells to proliferate and differentiate in mature osteoblasts, and have positive fusion outcomes in spinal indications [8,19,20].

We have observed that in high risk patients and smokers, this arthroscopic technique with minimal periosteal stripping and contribution of synthetic graft can improve the results.

## Conclusions

The use of bone graft biomaterials may reduce the chances of nonunion in tibiotalocalcaneal arthrodesis. Arthroscopic tibiotalocalcaneal arthrodesis can be implemented using i-FACTOR^(R)^Flex FR strip and has the potential to improve consolidation rates and provide early fusion.
